# Global climate and nutrient controls of photosynthetic capacity

**DOI:** 10.1038/s42003-021-01985-7

**Published:** 2021-04-12

**Authors:** Yunke Peng, Keith J. Bloomfield, Lucas A. Cernusak, Tomas F. Domingues, I. Colin Prentice

**Affiliations:** 1 Masters Programme in Ecosystems and Environmental Change, Department of Life Sciences, Imperial College London, Ascot, UK; 2 Department of Environmental Systems Science, ETH, Zurich, Switzerland; 3 Swiss Federal Institute for Forest, Snow and Landscape Research (WSL), Birmensdorf, Switzerland; 4 Department of Life Sciences, Imperial College London, Ascot, UK; 5 Centre for Tropical Environmental Sustainability Studies, James Cook University, Cairns, QLD Australia; 6 FFCLRP, Department of Biology, University of São Paulo, Ribeirão Preto, Brazil; 7 Department of Biological Sciences, Macquarie University, North Ryde, NSW Australia; 8 Department of Earth System Science, Tsinghua University, Beijing, China

**Keywords:** C3 photosynthesis, Ecological modelling

## Abstract

There is huge uncertainty about how global exchanges of carbon between the atmosphere and land will respond to continuing environmental change. A better representation of photosynthetic capacity is required for Earth System models to simulate carbon assimilation reliably. Here we use a global leaf-trait dataset to test whether photosynthetic capacity is quantitatively predictable from climate, based on optimality principles; and to explore how this prediction is modified by soil properties, including indices of nitrogen and phosphorus availability, measured in situ. The maximum rate of carboxylation standardized to 25 °C (*V*_cmax25_) was found to be proportional to growing-season irradiance, and to increase—as predicted—towards both colder and drier climates. Individual species’ departures from predicted *V*_cmax25_ covaried with area-based leaf nitrogen (*N*_area_) but community-mean *V*_cmax25_ was unrelated to *N*_area_, which in turn was unrelated to the soil C:N ratio. In contrast, leaves with low area-based phosphorus (*P*_area_) had low *V*_cmax25_ (both between and within communities), and *P*_area_ increased with total soil P. These findings do not support the assumption, adopted in some ecosystem and Earth System models, that leaf-level photosynthetic capacity depends on soil N supply. They do, however, support a previously-noted relationship between photosynthesis and soil P supply.

## Abstract (web summary)

Yunke Peng et al. use in-situ measurements and leaf trait data at 266 global sites for 1637 species and find that the maximum rate of carboxylation standardized to 25 °C is proportional to growing-season irradiance, and covaries with area-based leaf nitrogen and area-based phosphorus on the species level. These results challenge the assumption that leaf-level photosynthetic capacity depends on soil N supply yet supports the relationship between photosynthesis and soil P supply.

## Introduction

Accurate representation of photosynthetic capacity is critical for modelling the response of terrestrial ecosystems to environmental change^1,2^. Earth System models use the FvCB biochemical model^3^ to simulate responses of C_3_ photosynthesis to environment. The modelled instantaneous carbon-assimilation rate is limited either by *V*_cmax_ (μmol m^–2^ s^–1^), the maximum rate of carboxylation, or *J*, the light-dependent electron transport rate, which is asymptotic at high light towards *J*_max_ (μmol m^–2^ s^–1^). Both assimilation rates depend on temperature and on the intercellular partial pressure of CO_2_ (*C*_i_).

Application of the FvCB model^3^ requires knowledge of three ‘plant-determined’ quantities: *V*_cmax_, *J*_max_ and the ratio of *C*_i_ to the ambient partial pressure of CO_2_ (*C*_a_). This ratio, here called *χ*, is regulated by stomata. *J*_max_ and *V*_cmax_ are closely coordinated^4,5^. More data are available on *V*_cmax_ because it can be inferred from the light-saturated photosynthetic rate, which is commonly measured in the field^6^. Global models have to contend with the large observed variation (in time and space, and within and between species) of *V*_cmax_. Data analyses have explored its relationship to leaf nutrients^7–9^ and environmental variables^10,11^. Until recently, however, most models have assigned constant values of *V*_cmax_ at standard temperature (conventionally 25 °C: thus *V*_cmax25_) for each of a small number of plant functional types (PFTs), and allowed the temperature-dependent values to follow standard (instantaneous) equations of enzyme kinetics. Models also have to represent the plant-type and environmental dependencies of *χ* (ref. ^12^). Most models assign constant per-PFT values of parameters in one of the two widely used models for the response of stomatal conductance to vapour pressure deficit (*D*). However, these simplifications are not the best possible. *V*_cmax25_ and *χ* commonly vary at least as much within as between PFTs; while *χ* has predicted (and observed) relationships to growth temperature (*T*_g_) and to elevation above sea level (*z*) through its effect on atmospheric pressure, which are neglected in the standard models^10^.

One strand of recent research has accordingly focused on a search for universal responses to environment, applicable to all (C_3_) plants. Eco-evolutionary optimality hypotheses^12–15^ have been invoked in recent efforts to derive general principles for the prediction of plant traits and productivity^10,11,16–18^. The least-cost hypothesis^12,19^ proposes that investments in transpiration capacity (maintaining the water transport pathway) and *V*_cmax_ are balanced so that photosynthesis is achieved at the lowest total cost in maintenance respiration of leaves and stems. Within this framework, *χ* varies over a limited range, consistent with tight regulation of the balance between water loss and carbon gain^12^. The hypothesis predicts that *χ* should decline with increasing *D*, decreasing *T*_g_ and increasing *z*. Each of these predictions is quantitatively supported by global compilations of *χ* values inferred from stable carbon isotope measurements in leaves^10,20,21^ and wood^22^. The coordination hypothesis provides a framework to predict *V*_cmax_ from physical environmental variables: irradiance (photosynthetic photon flux density, PPFD) and temperature and CO_2_ (ref. ^23^). The ‘strong form’^24^ of this hypothesis states that carboxylation and electron transport are co-limiting under typical daytime growth conditions, so that neither is in excess. *V*_cmax25_ is observed to increase with PPFD, *D* and *z* (refs. ^10,11,21^), and to decline with *T*_g_ (refs. ^24,25^). The coordination hypothesis predicts all these observations. The decline with *T*_g_ is predicted because less Rubisco (the key carboxylation enzyme) is required to support photosynthesis in warmer environments^24^. The increases with *D* and *z* are predicted because greater photosynthetic capacity is required to support a given rate of carbon assimilation at lower *χ* (ref. ^26^).

Positive relationships between photosynthetic capacities and leaf N (*N*_area_)^27,28^ and leaf P (*P*_area_)^29–32^ are also widely observed. Much leaf N is invested in Rubisco^33–36^. Leaf P is required inter alia for cell membranes, nucleic acid synthesis and for ATP and NADPH production^9,37^. The predictive power of relationships to *N*_area_ or *P*_area_ is often weak^11,38–40^; however, recent studies^8,9^ have proposed a framework in which *V*_cmax25_ is constrained by the lesser of two functions, one related to *N*_area_ and the other to *P*_area_. Leaf nutrient levels, in turn, may or may not reflect their availability in the soil. *N*_area_ can be related to soil pH (or fertility) but is not unambiguously related to soil N availability^14^, while *P*_area_ is related to both soil fertility and total soil P^14,41^.

Thus, there are two conflicting paradigms to explain worldwide variation in photosynthetic capacity. One emphasizes its predictability from climate, based on optimality principles. The other emphasizes its predictability from leaf nutrients. This second approach has been extended to embrace the assumption that leaf nutrients reflect soil nutrient availability—although this is not universally true^42^.

To help resolve this contradiction, we assembled a large global dataset of *V*_cmax25_, *N*_area_ and *P*_area_ data from multiple species and sites. In situ soil measurements (pH, C:N ratio and total P) were available at a subset of the sites. Rather than total soil N, which mainly relates to soil organic content, we used soil C:N as an inverse measure of N availability^43^. We hypothesized that

1. Photosynthetic capacity is subject to first-order control by climate, as predicted by the coordination and least-cost hypotheses. *V*_cmax25_ increases in proportion to PPFD and increases towards colder and drier environments, due to greater biochemical investment required when *χ* is low.
2. Photosynthetic capacity is reduced, compared to climate-based predictions, under conditions of low nutrient (N and/or P) availability.

## Results

**Fig. 1:**
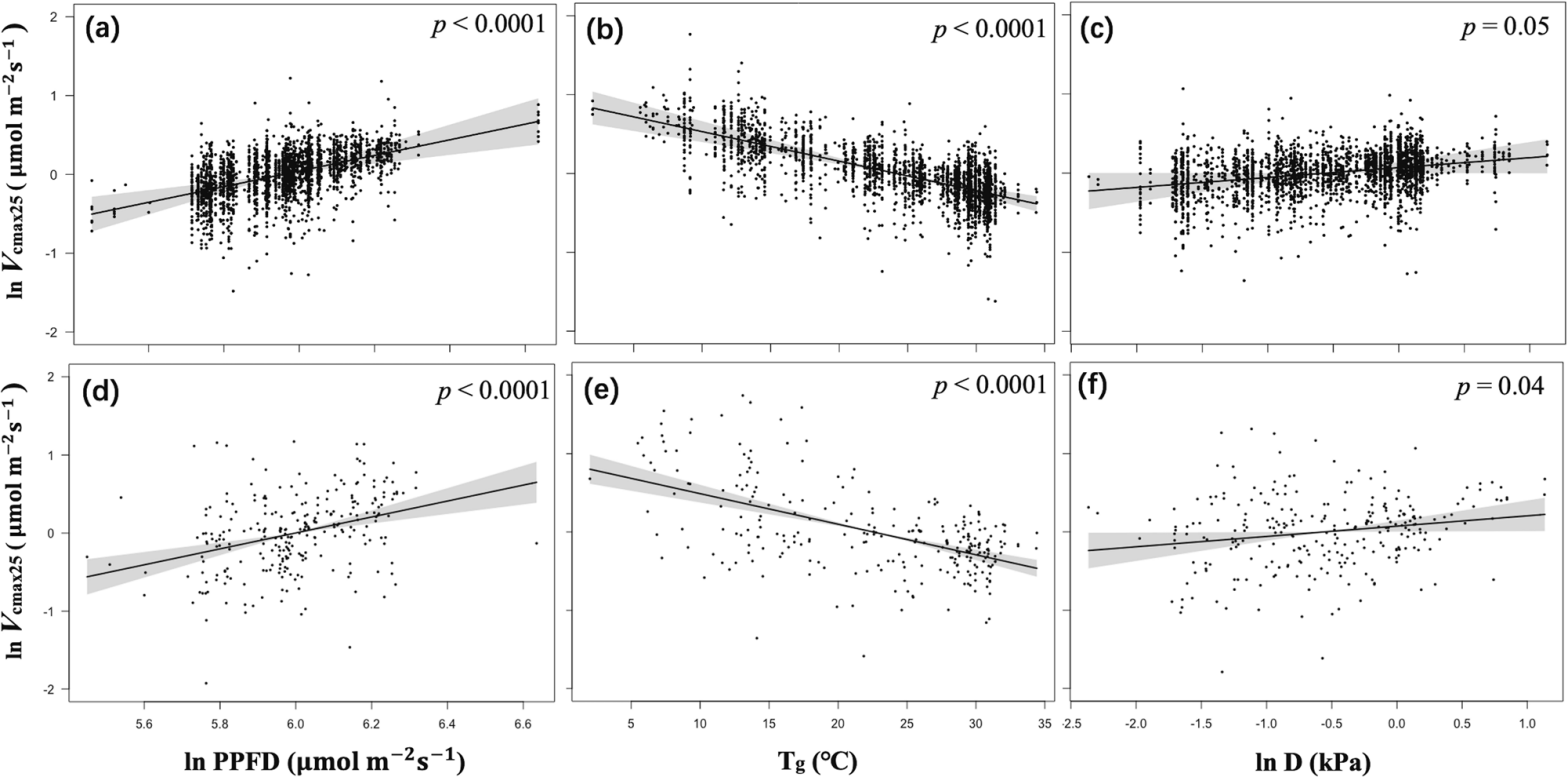
Partial residual plots for *V*_cmax25_ in relation to climate variables. Partial residual plots for log-transformed *V*_cmax25_: all-species (**a**, **b**, **c**) and site-means (**d**, **e**, **f**). Coefficients and standard errors for the fitted lines are given in Supporting Information Table S4.

Theoretically predicted values (see ‘Methods’) of the derivatives of ln *V*_cmax25_ against ln PPFD, *T*_g_ and ln *D* are given in Table 1, for comparison with values fitted by statistical models (Table 1, Fig. 1). The value of 1 for the derivative of ln *V*_cmax25_ with respect to ln PPFD implies proportionality, i.e. a 10% increase in PPFD induces a 10% increase in *V*_cmax25_. The value of –0.05 K^–1^ for the derivative of ln *V*_cmax25_ with respect to *T*_g_ implies that a 1 °C increase in growth temperature is predicted to induce a 5% decrease in *V*_cmax25_. Regression coefficients of *V*_cmax25_ against the same climate variables were statistically indistinguishable from theoretically predicted values (Table 1). Analysis of site-mean data explained more variance than a mixed-model analysis of all species (see ‘Methods’), indicating that a greater fraction of variation in photosynthetic capacity can be explained by physical environmental constraints when considering the whole community together, excluding variation within the community. The response of *V*_cmax25_ to *D* was slightly steeper in the ‘observed’ than the ‘theoretical’ relationship, but the difference was within one standard error. From the random term of the all-species mixed model (see ‘Methods’), species and site identity separately accounted for 22 and 50% of the variation in *V*_cmax25_ that was unexplained by the model’s climate variables (Table S1).

**Table 1:** Summary statistics for the climatic dependencies of *V*_cmax25_ (μmol m^–2^ s^–1^).

Predictor for *V*_cmax25_	Theoretical value	All-species coefficient *R*^2^ = 0.17	Site-mean coefficient *R*^2^ = 0.31
ln PPFD	1	0.99 ± 0.22	1.02 ± 0.21
*T*_g_	–0.05 K^–1^	–0.04 ± 0.01 K^–1^	–0.04 ± 0.01 K^–1^
ln *D*	0.07	0.13 ± 0.06	0.13 ± 0.06

Log-transformed photosynthetic capacities standardized to 25  °C were derived for all species and as site means. Theoretical values were obtained by evaluating partial derivatives of Eq. (3) with respect to each variable at the median climate of the global dataset (PPFD = 400 μmol m^–2^ s^–1^, *T*_g_ = 25  °C, *D* = 0.60 kPa). All-species coefficients represent the partial effects of each variable, estimated in a mixed effects model with site and species as random effects. Site-mean coefficients represent the partial effect of each variable, estimated in a fixed effects model. All fitted values are given ±1 standard error.

**Fig. 2:**
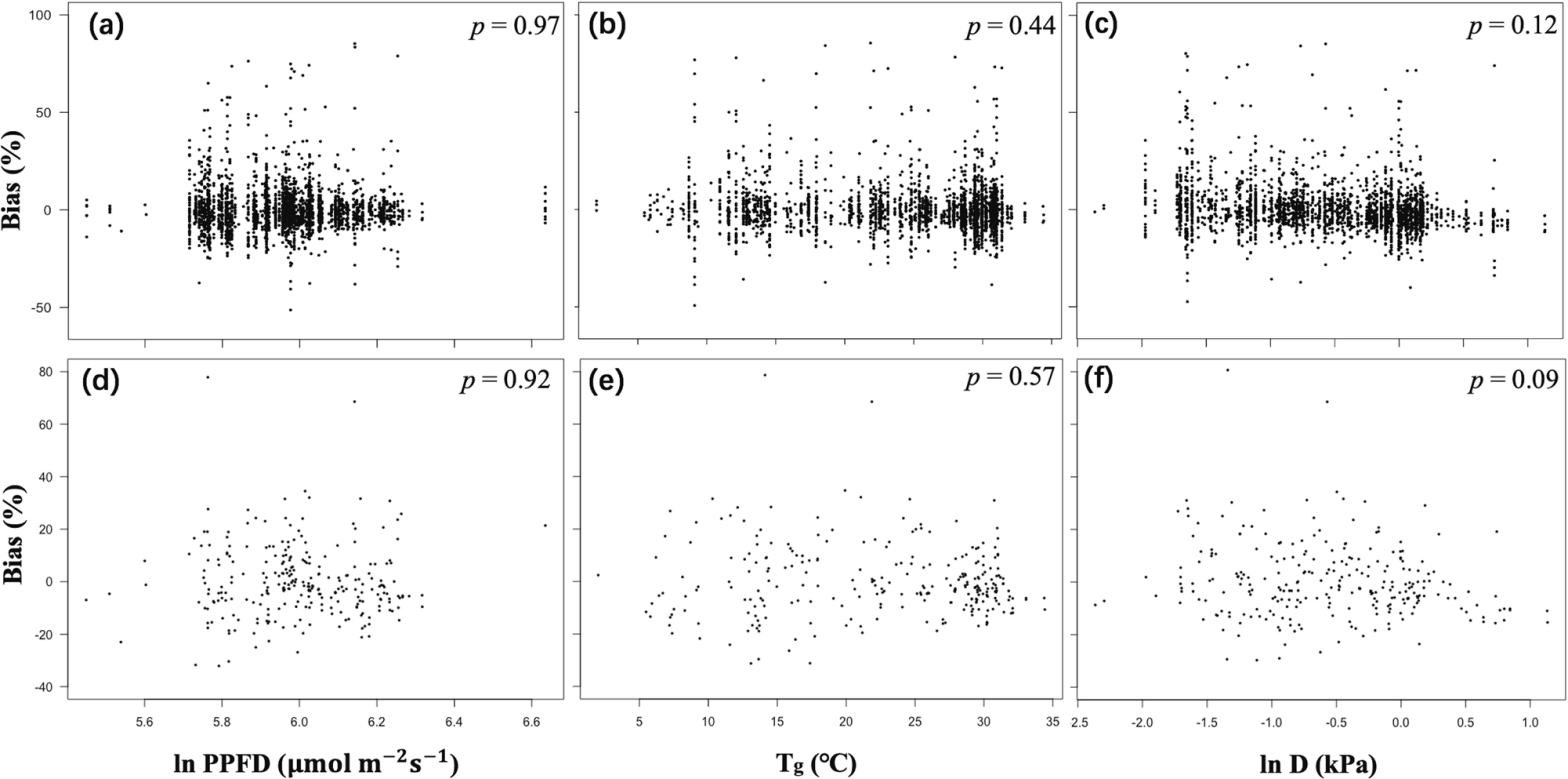
Partial residual plots for the model bias of theoretically predicted *V*_cmax25_ values in relation to climate variables. Partial residual plots for the model bias of theoretically predicted *V*_cmax25_ values in relation to climate variables: all-species (**a**, **b**, **c**) and site means (d, e, f). Coefficients and standard errors for the fitted lines are given in Supporting Information Table S4.

No significant bias was shown for the predicted relationship of *V*_cmax25_ to PPFD, *T*_g_ or *D* (Fig. 2). There was a possible underestimation of *V*_cmax25_ at higher *D*, but this trend was not significant either in all-species (Fig. 2c; *p* = 0.12) or site-mean (Fig. 2f; *p* = 0.09) analyses.

**Fig. 3:**
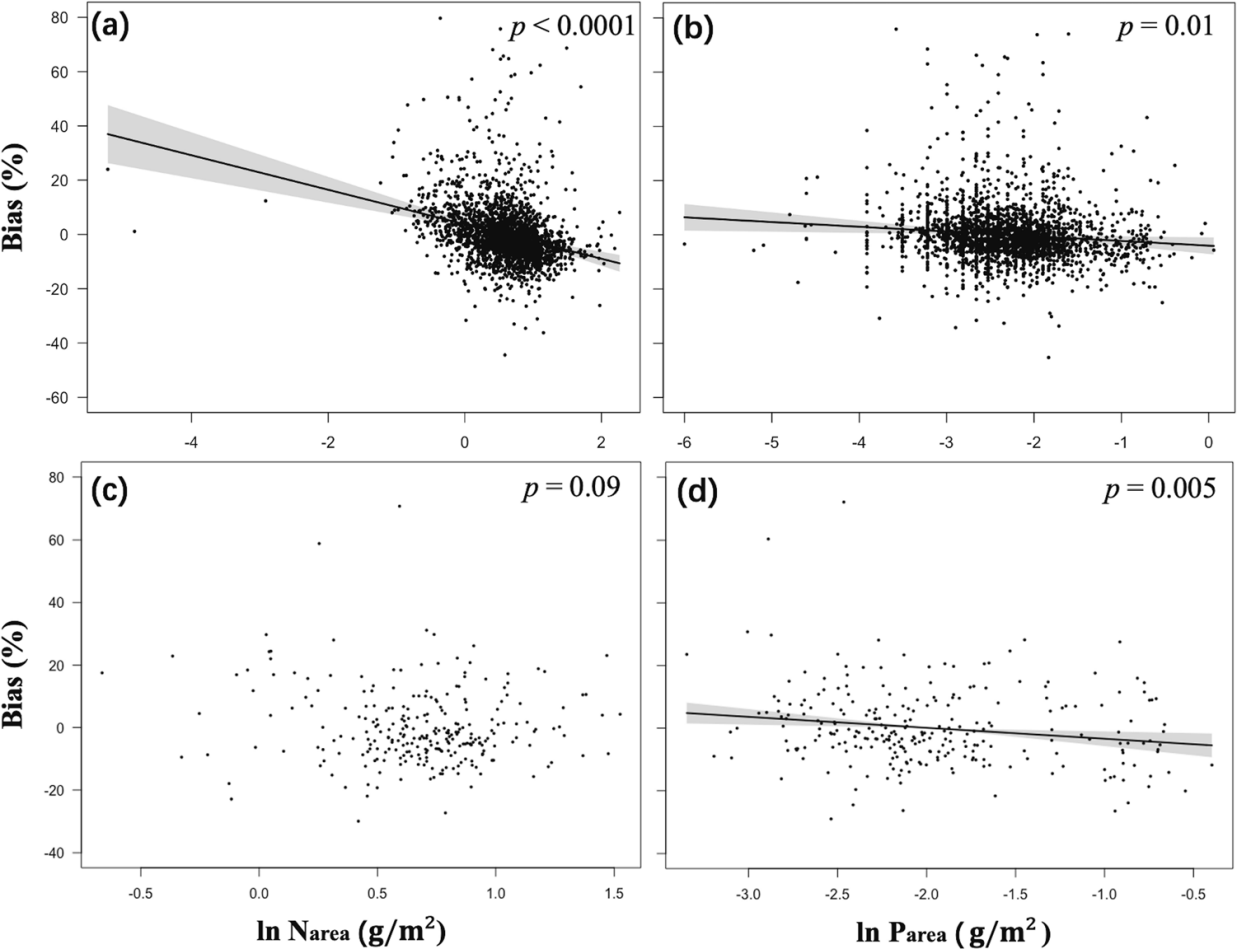
Partial residual plots for the model bias of statistically fitted *V*_cmax25_ in relation to leaf nutrients. Partial residual plots for the model bias of statistically fitted *V*_cmax25_ (Table 1) in relation to leaf nutrients, for all-species (**a**, **b**) and site-mean (**c**, **d**) data. The model bias represents the difference between predicted and observed *V*_cmax25_, where the predicted *V*_cmax25_ was based on the climate-driven regressions fitted from site-mean and all-species data as shown in Table 1. Coefficients and standard errors for the fitted lines are given in Supporting Information Table S4.

Statistical models of photosynthetic capacity (all species and site means) as a function of climate overestimated *V*_cmax25_ in low-P leaves and underestimated *V*_cmax25_ in high-P leaves (Fig. 3b, d). The all-species statistical model also showed a bias in *V*_cmax25_ related to leaf N (Fig. 3a). This relationship was still apparent (*p* < 0.0001) after removal of three highly influential points. The three species with extremely low *N*_area_ values (*Turpinia pomifera, Uncaria laevigata* and *Walsura pinnata*) shown in Fig. 3a were sampled in Yunnan, China (21.6°N, 101.5°E). These species possessed very low *V*_cmax25_ (21 μmol m^–2^ s^–1^) values, probably a consequence of growth in deep shade. In contrast to the all-species model, the site-mean model showed no bias with respect to *N*_area_ (Fig. 3c).

**Fig. 4:**
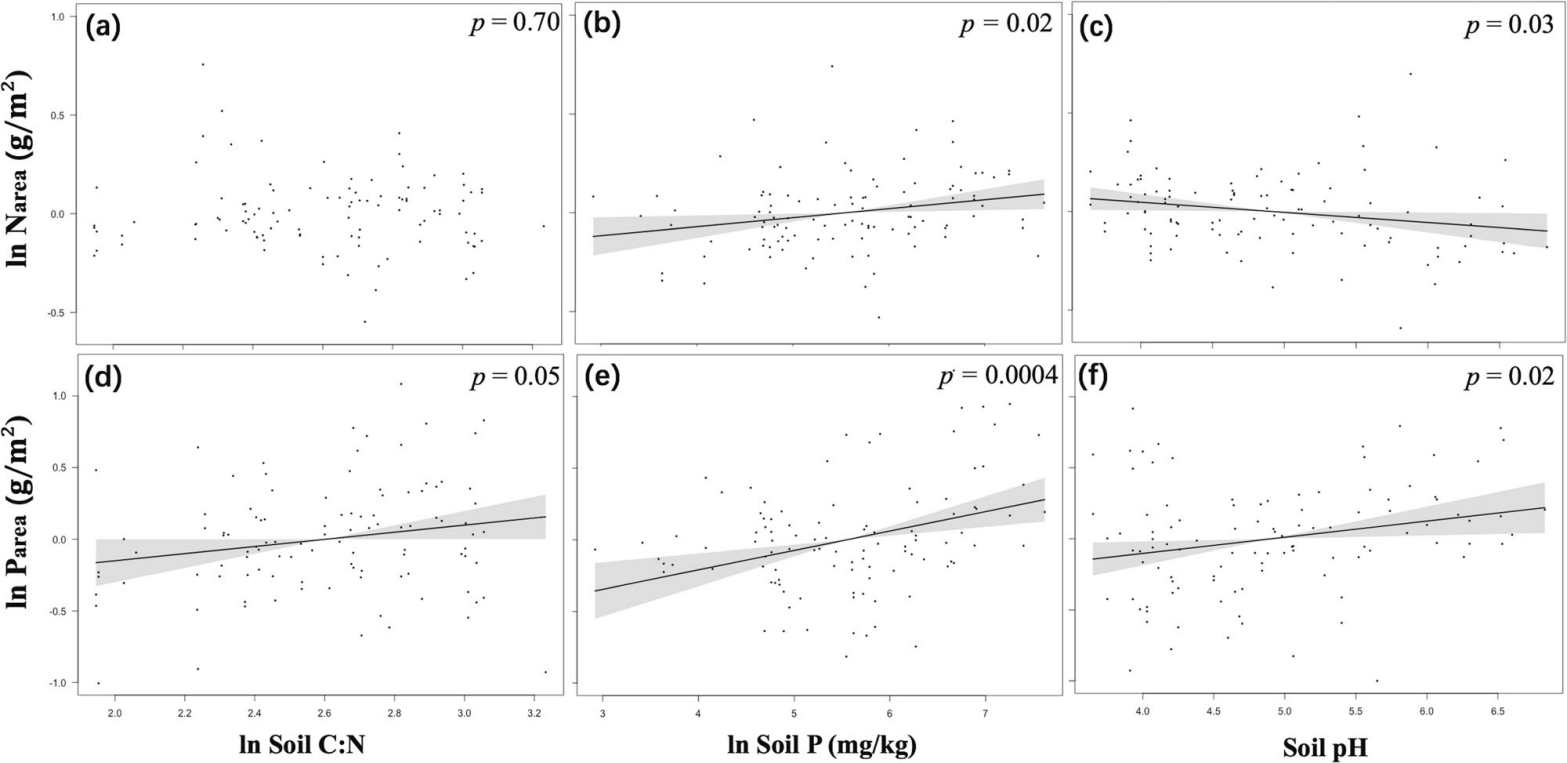
Partial residual plots for leaf nutrients in relation to in situ measured soil properties. Partial residual plots for leaf nutrients (site means) in relation to in situ measured soil properties, for Narea (**a**, **b**, **c**) and Parea (**d**, **e**, **f**) data. Analyses for all species are shown in Fig. S1. Coefficients and standard errors for the fitted lines are given in Supporting Information Table S4.

Analysis of the subset of the data with in situ soil measurements indicated that *P*_area_ increased with soil C:N ratio, total soil P and soil pH (Figs. 4d–f, S1d–f). *N*_area_ increased with soil P (Figs. 4b, S1b) and decreased with soil pH (Figs. 4c, S1c). No relationship was found between leaf N and soil C:N ratio (Figs. 4a, S1a).

**Fig. 5:**
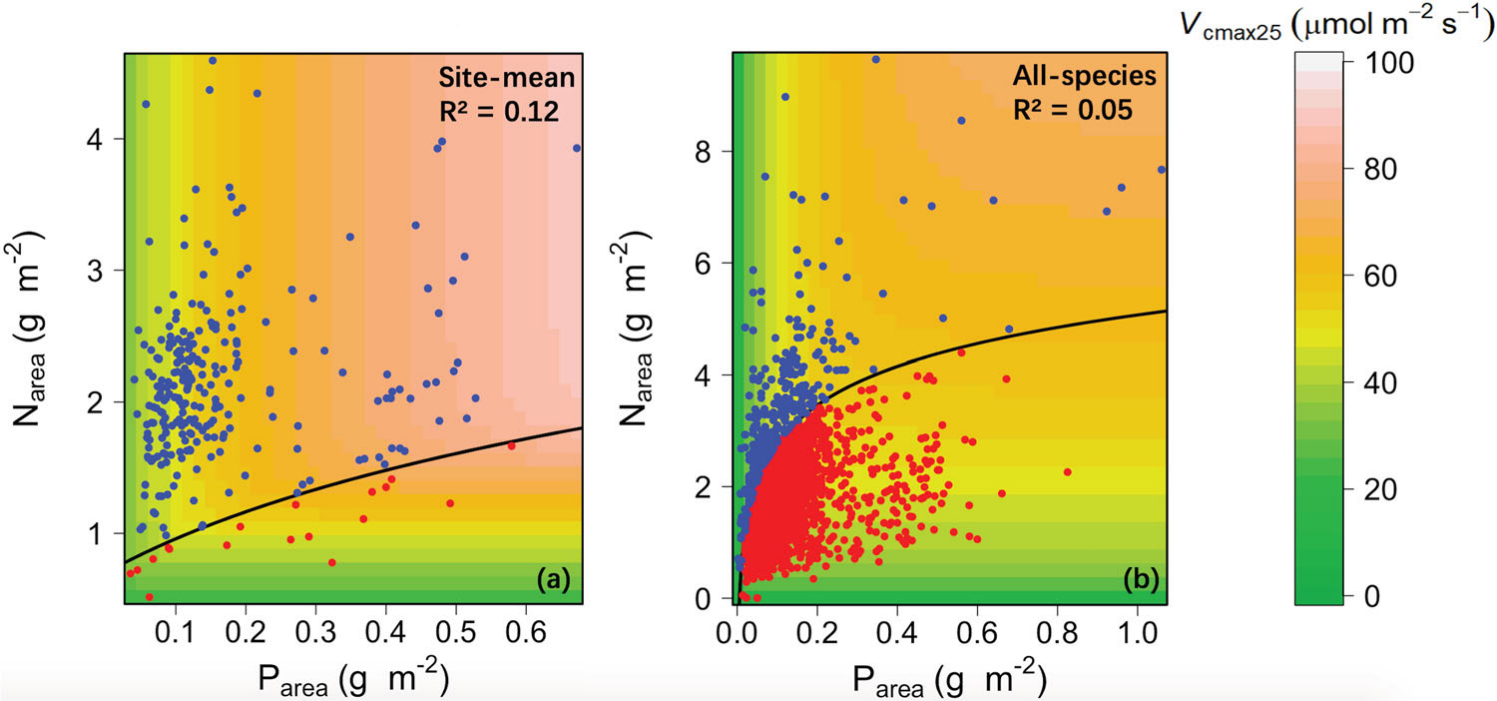
Visualizing the co-limitation of *V*_cmax25_ by Narea and Parea based on the minimum function model. Visualizing the co-limitation of *V*_cmax25_ by Narea and Parea for global (**a**) site-mean and (**b**) all-species analyses, based on the minimum function model. Following Domingues et al.8, blue points represent cases where Parea was the ‘limiting’ nutrient; red points represent cases where Narea was the ‘limiting’ nutrient. The fitted regression line in (**a**) is Narea = ln (5.96 Parea + 2.01) and in (**b**) is Narea = ln (158.62 Parea + 0.11).

Global *V*_cmax25_ could, alternatively, be represented by a minimum function (Eq. 12) of *N*_area_ and *P*_area_. This function provided a better fit to the data than linear regression models with *V*_cmax25_ as a function of *N*_area_ and *P*_area_ and combinations thereof or a model including *N*_area_, *P*_area_ and their interaction (Table S2). In site-mean analysis based on the minimum function *P*_area_ was shown to be the principal limiting factor (93% of sites). In all-species analysis, *N*_area_ was shown to be the principal limiting factor (86% of species; Fig. 5). This contrast agrees with our findings for model bias: *V*_cmax25_ variations within sites are more related to leaf N, while variations between sites (community means) are related to mean leaf P but not to mean leaf N. However, the goodness of fit of these models based on nutrients alone (*R*^2^ = 0.05, 0.12 for all species and site mean, respectively) was inferior to that of models based on climate alone (*R*^2^ = 0.17, 0.31).

## Discussion

The optimality framework accounts for the major global patterns of photosynthetic capacity as shown in our dataset. Consistent with hypothesis (1), global patterns of *V*_cmax25_ were found to be predictable to first order from PPFD, growth temperature and vapour pressure deficit. Proportionality to PPFD is consistent with observations on light gradients^44^, seasonal dynamics^45^ and the cloud immersion effect, which decreases PPFD and *V*_cmax25_ at mid elevations of tropical mountains^39^. *V*_cmax25_ was predicted (and found) to be greater in drier environments: consistent with the larger biochemical investment required to achieve optimal photosynthesis when stomata are more closed. We found a somewhat steeper than predicted response to *D* and thus a slight but non-significant underestimation of *V*_cmax25_ at higher *D*. This might be because the least-cost hypothesis does not consider the compounding effect of low soil moisture, which often accompanies high *D* and further decreases stomatal conductance, therefore preventing excessive transpiration but increasing the investment in carboxylation capacity^22,46,47^. In short-term drying experiments, *V*_cmax25_ typically declines steeply (at different critical pre-dawn water potential values dependent on species^48,49^), although an increase in leaf-level *V*_cmax25_—which may be accompanied by a reduction in leaf area—can be observed when plants are allowed to acclimate to moderate drought^50–55^. These findings are consistent with the expectation^56^ that a decrease of *V*_cmax_ under drought conditions is linked to a declining hydraulic capacity of the soil–root–xylem system, which can be accommodated over time by leaf shedding. *V*_cmax25_ showed a negative response to growth temperature, which is predicted because greater investment in photosynthetic enzymes is required at lower temperatures to produce the same catalytic activity^10,55^. Thermal acclimation according to this optimality principle is supported by evidence for a decline of light use efficiency^57^ and an increase of photosynthetic nitrogen use efficiency^58^ towards warmer environments, and by increased *V*_cmax25_ at higher elevations^21,41^. The percentage variance explained by these relationships is modest, however (31% for site-mean data: Table 1), consistent with findings by van der Plas et al.^59^ on the limits to predictability of ecosystem function from plant traits.

Our hypothesis (2) is partially supported by the analysis of bias in the statistically fitted model. Consistent with findings by Maire et al.^14^, we showed an overestimation of *V*_cmax25_ in leaves with low *P*_area_. These are typical of sites on acid soils and/or low soil P availability, including some wet tropical forests^14,39^. Many tropical soils are characterized by low total soil P due to long-term weathering^60,61^, and a dependency of net primary production on P availability has been shown in tropical forests^62^. Small-scale experimental studies have also suggested that low soil P availability can decrease the light-saturated photosynthetic rate (*A*_sat_)^63–65^ and *V*_cmax_^66,67^. Adaptation strategies to cope with long-term P deficiency include restricting export of triose phosphate to the cytosol^68^, preventing the phosphorylation of ADP to ATP^37,69^, phosphate recycling during photorespiration^70^ and the replacement of phospholipids by galactolipids and sulpholipids^71,72^, all potentially entailing additional costs to the plant. On the other hand, photosynthesis in tropical forests is typically not limited by N^73^.

The global relationship between *V*_cmax25_ and *N*_area_^74^ primarily reflects the large amount of N invested in Rubisco and other photosynthetic enzymes^75^. Leaves with a high photosynthetic capacity necessarily have a large N content per unit area. Within vegetation canopies, *V*_cmax25_ and *N*_area_ both vary greatly, especially along the light gradient from the canopy top to the understory—as shown in many empirical studies^23,35,76,77^ and further discussed elsewhere^78,79^. Our data provide no information on the range of light environments within sites and, therefore, our finding of a bias (low-N leaves having lower than predicted *V*_cmax25_) in the all-species analysis is no surprise. However, the relationship disappeared in the site-mean analysis, indicating that *V*_cmax25_ at community level is predictable without the need to consider leaf N. Moreover, we found no support for the hypothesis (assumed in some ecosystem and Earth System models) that leaf N is determined by soil N availability—suggesting that the metabolic component of leaf N is determined by photosynthetic capacity, as proposed by Dong et al.^28^, rather than vice versa. We did however find that leaf N increases with soil P, which is consistent with the observed effect of soil P on photosynthetic capacity.

A limitation of our analysis is its implicit assumption that mesophyll conductance (*g*_m_) is not limiting to photosynthesis. *V*_cmax_ as estimated here, therefore, is an ‘apparent’ value and likely to underestimate the true photosynthetic capacity by a variable amount, which cannot be predicted from data currently available at a large scale. However, this simplification reflects the situation in the great majority of ecosystem models, and it has been indicated that ‘greater process knowledge of *g*_m_ will be required before it can be included [in models]’ (ref. ^80^, p. 26). A more comprehensive understanding of the relationships between leaf nutrients and photosynthesis will depend on advances in understanding the anatomical and physiological controls of *g*_m_ (refs. ^81,82^), and extensions of leaf-level optimality theory to consider these controls.

In conclusion, while the short-term control of photosynthesis is relatively well understood (and modelled), the longer-term control of photosynthetic capacity is different, and subject to conflicting interpretations. Our findings show that the first-order climatic controls of *V*_cmax25_ are relatively strong and predictable, indicating that models must account for them. Our results are not consistent with the model assumption that soil N availability controls leaf N, which in turn controls *V*_cmax25_. They are, however, consistent with previous observational and experimental results indicating the existence of P limitation on leaf P, leaf N and *V*_cmax25_.

## Methods

During photosynthesis, *C*_i_ declines relative to *C*_a_ because C assimilation removes CO_2_ from the intercellular spaces while the stomata impose a resistance to the diffusion of CO_2_ into the leaf from the air. The *C*_i_/*C*_a_ ratio (*χ*) is maintained within a limited range (about 0.5–0.9 in C_3_ plants) that is determined by the growth environment^83^. According to the least-cost hypothesis^12,19^, *χ* is controlled by stomata in such a way as to minimize the sum of the unit costs of the required capacities for transpiration and carboxylation. A consequence of this hypothesis is that for any given set of environmental conditions, there is an optimal value of *χ*^10,12^

1 $${\upchi}_{{\mathrm{opt}}} = \frac{{{\Gamma} \ast }}{{C_a}} + \frac{{( {1-\frac{{{\Gamma} \ast }}{{C_a}}} )\xi }}{{\xi + \sqrt D }},{\mathrm{where}}\;\xi = \sqrt {\left[ {\frac{{\beta \left( {K + {\Gamma} \ast } \right)}}{{1.6\;\eta \ast }}} \right]}$$

that satisfies the least-cost criterion. Here, Γ* is the photorespiratory compensation point, i.e. the value of *C*_i_ at which gross photosynthesis is zero; *Κ* is the effective Michaelis–Menten coefficient of Rubisco (Pa); *D* is the leaf-to-air vapour pressure deficit (Pa); η* is the viscosity of water relative to its value at 25 °C and β is the ratio of the unit costs of maintaining carboxylation and transpiration activities at 25 °C, estimated as 146 based on a global compilation of leaf stable carbon isotope measurements^10^. *K* is given by

2 $$K = K_{\mathrm{C}}\left( {1 + O/K_{\mathrm{O}}} \right)$$

where *K*_C_ and *K*_O_ are the Michaelis–Menten coefficients of Rubisco for CO_2_ and O_2_, respectively (Pa, reflecting the twin affinities of Rubisco), and *O* is the partial pressure of O_2_ (Pa). Γ*, *Κ*_C_ and *K*_O_ are functions of temperature, which we apply based on in vivo measurements on tobacco plants^84^. Γ*, *C*_a_ and *O* also vary with elevation, in direct proportion to atmospheric pressure.

The coordination hypothesis states that under typical daytime growth conditions photosynthesis is co-limited by carboxylation and electron transport. Optimal *V*_cmax_ is calculated as

3 $$V_{{\mathrm{cmax}},\;{\mathrm{opt}}} = \varphi _0I_{{\mathrm{abs}}}[ {( {C_{\mathrm{i}} + K} )/( {C_{\mathrm{i}} + 2{\Gamma}^ \ast } )} ]$$

where φ_0_ is the intrinsic quantum efficiency of photosynthesis (mol C mol^–1^ photons); *I*_abs_ is the PPFD absorbed by the leaf (μmol photons m^–2^ s^–1^). These values were corrected to 25°C using the Arrhenius equation with activation energies from Bernacchi et al.^84,85^. Intrinsic quantum efficiency was assumed to follow the temperature dependency of electron transport in light-adapted leaves^85^

4 $$\varphi _0 = (0.352 + 0.021\;T_{\mathrm{g}}-3.4 \times 10^{-4}T_g^2)/8$$

According to Eq. (3) and its derivatives, optimal *V*_cmax_ increases in proportion to PPFD. It also increases with *T*_g_. On the other hand, optimal *V*_cmax25_ *declines* with *T*_g_. This is because the enzyme-kinetic effect, leading to a reduced *V*_cmax25_ requirement at higher temperatures (caused by the temperature dependency of Rubisco activity), is stronger than the photorespiratory effect, leading to an increased *V*_cmax_ requirement at higher temperatures (caused by the temperature dependencies of *K* and Γ*). Experimental manipulations of growth temperature^86^, repeated measurements on the same plants at different seasons^24^, global spatial patterns of *V*_cmax_^11^ and variations of *V*_cmax25_ on a long elevation transect^41^ are all consistent with the negative temperature dependency of *V*_cmax25_ implied by Eq. (3).

Quantitative predictions of the effect of each climate variable on ln *V*_cmax25_ can be obtained by taking partial derivatives of Eq. (3) with respect to each variable in turn^21^. Logarithmic transformation is appropriate for magnitude variables described by multiplicative expressions like these^87^. The theory predicts approximately linear relationships of ln *V*_cmax25_ to ln PPFD, ln *D* and (without transformation) *T*_g_^21^. These derivatives were evaluated at the median climate of the dataset (PPFD = 400 μmol m^–2^ s^–1^, *T*_g_ = 25°C, *D* = 0.60 kPa) using the deriv package in R (ref. ^88^) (Table 1).

### Photosynthetic data

The leaf-trait dataset comprised measurements at 266 sites for a total of 1637 species and 5000 individuals, and soil measurements for 39% of sites (Fig. S2). The dataset consists of field measurements made in natural (unfertilized) vegetation, from several published data sources^7,8,14,20,28,73,89–94^. The numbers of species recorded within each PFT (ref. ^95^) are provided in Table S3. *V*_cmax_ values were derived either from CO_2_ response (*A*–*C*_i_) curves (94% of the dataset) or the one-point method^6^ from single measurements of light-saturated net photosynthesis (*A*_sat_) (6% of the dataset). The one-point method provides a way to estimate *V*_cmax_ knowing only *A*_sat_, day respiration (*R*_d_), temperature and atmospheric pressure

5 $$V_{{\mathrm{cmax}}}\left[ {{\mathrm{est}}} \right] \approx \left( {A_{{\mathrm{sat}}} + R_{\mathrm{d}}} \right)\left( {C_{\mathrm{i}} + K} \right)/( {C_{\mathrm{i}}-{\Gamma}^ \ast } ).$$

If no respiration measurement was available, the following approximation was used instead

6 $$V_{{\mathrm{cmax}}}\left[ {{\mathrm{est}}} \right] \approx A_{{\mathrm{sat}}}/[ {( {C_{\mathrm{i}}-{\Gamma}^ \ast } )/( {C_{\mathrm{i}} + K} )-0.015} ]$$

where *R*_d_ is assumed to be 1.5% of *V*_cmax_^6,40,96^. Rogers et al.^97^ indicated that the one-point method could result in a twofold underestimation of photosynthetic capacity in the Arctic region. Burnett et al.^98^ however estimated errors in photosynthetic capacity at around 20% at most, suggesting that *V*_cmax_ data obtained in this way (which, in any case, constitute only a small fraction of the dataset) can be justified in the context of a global survey. If measurements were made at a temperature other than 25 °C, reported *V*_cmax_ and *J*_max_ values were standardized to 25°C using activation energies provided by Bernacchi et al.^84,85^.

### Climate data

Monthly average values of mean daily maximum (*T*_max_, °C) and minimum (*T*_min_, °C) temperatures were extracted at the 0.5° grid location of each site from Climate Research Unit data (CRU TS 4.01)^99^, either for the measurement year or for the period 1991–2010 at sites not reporting measurement year. These data were three-dimensionally interpolated to actual site locations (longitude, latitude, elevation) using Geographically Weighted Regression (GWR) in ArcGIS. Mean daytime air temperature (*T*_g_) was estimated for each month by assuming the diurnal temperature cycle to follow a sine curve, with daylight hours determined by latitude and month

7 $$T_g = T_{{\mathrm{max}}}\left\{ {1/2 + \left( {1-x^2} \right)^{1/2}/2\;\cos ^{-1}x} \right\} + T_{\min }\left\{ {1/2-\left( {1-x^2} \right)^{1/2}/2\;\cos ^{-1}x} \right\},\;x = -\tan \lambda \tan \;\delta$$

where *λ* is latitude and *δ* is the monthly average solar declination^100^. Monthly values of *T*_g_ were averaged over the thermal growing season, i.e. months with mean daily temperature > 0 °C.

Incident solar radiation data were derived from WATCH Forcing Data ERA-Interim^101^ at the same period and resolution, and also interpolated by GWR. Solar radiation (W m^–2^) was converted to PPFD by multiplication by the energy-to-flux conversion factor 2.04 (μmol J^–1^)^102^. PPFD was averaged across the thermal growing season. Mean atmospheric pressures (*P*_atm_) were derived using the barometric formula^102,103^. *D* (kPa) was estimated using the Magnus–Tetens formula^46^

8 $$D = e_{\mathrm{s}}-e_{\mathrm{a}},$$

with

9 $$e_{\mathrm{s}} = 0.611\;\exp \;[17.27\;T/(T + 237.3)],\;{\mathrm{where}}\;T = \left( {T_{\min } + T_{\max }} \right)/2$$

and

10 $$e_{\mathrm{a}} = \left[ {P_{{\mathrm{atm}}}W_{{\mathrm{air}}}R_{\mathrm{v}}} \right]/\left[ {R_{\mathrm{d}} + W_{{\mathrm{air}}}R_{\mathrm{v}}} \right]$$

where *W*_air_ is the mass mixing ratio of water vapour to dry air; *W*_air_ = *q*_air_ / (1 – *q*_air_), where *q*_air_ is the specific humidity (kg/kg) derived from WATCH Forcing Data ERA-Interim^101^, *R*_d_ and *R*_v_ are the specific gas constants of dry air and water vapour, *R*_d_ = *R*/*M*_d_ and *R*_v_ = *R*/*M*_v_, where *R* is the universal gas constant (8.314 J^–1^ K^–1^), *M*_d_ is the molar mass of dry air (28.963 g mol^–1^) and *M*_v_ is the molar mass of water vapour (18.02 g mol^–1^).

### Statistical analysis

The climate data were used to make theoretical predictions of relationships between photosynthetic capacity and climate variables based on the optimality framework, and independently, to derive statistical relationships by multiple regression (Tables S2 and S4). Separate statistical analyses were carried out for individual species, and for site-averaged measurements. In the analyses of individual species (i), each data-point represents the average of one or more measurements on a particular species at a site (*n* = 2513). In the analyses of site-averaged measurements (ii), each data-point represents an average for a site (across all individual and species; *n* = 266) (Table 1). Analyses of type (i) (‘all species’) data were carried out by means of a linear mixed effects model using the nlme package in R^88^. Climate variables (*T*_g_, *D*, PPFD) were included as fixed terms, with site and species as random intercepts. A crossed rather than a fully nested random design was used because some species occurred at more than one site. Ordinary least squares multiple linear regression, using the lm function in R^88^, was used for analyses of type (ii) (‘site mean’) data. Regression relationships were visualized using partial residual plots, obtained with the visreg package in R^88^. Partial residual plots display the relationship between values of the response variable versus each predictor variable, after those responses have been adjusted to hold all other predictors constant at their median values in the dataset. Photosynthetic capacities, PPFD and *D* were natural log-transformed before analysis so that the resulting regression coefficients can be directly compared with theoretical predictions (Table 1).

### Model data comparisons

Model bias (*B*, %) in *V*_cmax25_ was calculated as follows:

11 $$B = 100\left( {V_{{\mathrm{cmax}}25}\left[ {{\mathrm{pred}}} \right]-V_{{\mathrm{cmax}}25}\left[ {{\mathrm{obs}}} \right]} \right)/V_{{\mathrm{cmax}}25}\left[ {{\mathrm{obs}}} \right]$$

where *V*_cmax25_[pred] is a predicted value and *V*_cmax25_[obs] an observed value. Using theoretically predicted values, we explored whether *B* was significantly related to the climate variables. If so, this would indicate that the true responses of *V*_cmax25_ to climate variables were different from the predicted ones—pointing to something missing (or wrong) in the theory. Then, we explored whether bias in the values predicted by the statistical models (both all-species and site-mean models) was significantly related to leaf *N*_area_ and *P*_area_. If found, such bias would indicate effects of leaf nutrients, additional to the effects of the climate variables considered.

### Alternative models for the response to leaf nutrients

An alternative statistical model for photosynthetic capacity is a ‘minimum function’ of *N*_area_ or *P*_area_^8^. The following differentiable equation is almost exactly equivalent to a minimum function (Fig. S3):

12 $$Z = -\left( {1/k} \right)\;\ln \;\left[ {e^{-kx} + e^{-ky}} \right]$$

where *Z* is the response variable (*V*_cmax25_), *x* and *y* are the predictor variables (*N*_area_, *P*_area_) and *k* ≫ 1. Equation (12) is the ‘log-sum-exp’ formula, which provides a continuous approximation to the minimum function—allowing its use in regression, and comparison of goodness-of-fit statistics with ordinary linear regression (Table S2). The larger the value of *k*, the closer the approximation to the minimum function. A simple sensitivity analysis showed that large values of *k* (≥10) gave best performance (Table S5), indicating that the minimum function fitted the data better than a smooth transition between N and P limitation. Equation (12) was fitted to both all-species and site-mean data (Fig. 5). The equation was plotted using an iterative least squares procedure using the akima, stats and grDevices packages in R^88^.

### Statistics and reproducibility

Data collection, formulae and statistical analyses are described in ‘Methods’. All statistical analyses used R software (ref. ^88^), applying ordinary linear regression for site-mean analysis and a mixed effects model for all-species analysis. All R packages applied are referenced in ‘Methods’. The relevant statistics for the main analyses are presented in Supplementary Information.

### Reporting summary

Further information on research design is available in the Nature Research Reporting Summary linked to this article.

## Supplementary information

Peer Review File

Supplementary Information

Reporting Summary

## Data Availability

No new data were collected for this analysis. The photosynthesis, leaf-trait and soils data are available from the authors of papers cited in the ‘Methods’ section^7,8,14,20,28,73,89–94^. The complete photosynthesis, climate, leaf-trait and soils datasets underlying all analyses are also publicly available at Zenodo^104^ and GitHub: https://github.com/yunkepeng/VcmaxMS. In case of any issues concerning the observed and predicted data and for all queries on ancillary information including the climate data, please contact Y.P. (yunke.peng@usys.ethz.ch) or C.P. (c.prentice@imperial.ac.uk).
