# Supplementary material for: Global climate and nutrient controls of photosynthetic capacity
Source: Commun Biol. 2021 Apr 12;4:462. doi: 10.1038/s42003-021-01985-7 (PMC8042000; doi:10.1038/s42003-021-01985-7)
Supplement: Supplementary file 2 — Supplementary Information [file 42003_2021_1985_MOESM2_ESM.pdf]

Supplementary Information (Figs S1-S3; Tables S1-S5)

Global climate and nutrient controls of photosynthetic capacity

Yunke Peng[1,2,3], Keith J. Bloomfield[4], Lucas A. Cernusak[5], Tomas F. Domingues[6] and I. Colin Prentice[4,7,8]

[1]Masters Programme in Ecosystems and Environmental Change, Imperial College London, Department of Life Sciences, Silwood Park Campus, Buckhurst Road, Ascot SL5 7PY, UK
[2]Department of Environmental Systems Science, ETH, Universitätsstrasse 2, 8092 Zurich, Switzerland
[3]Swiss Federal Institute for Forest, Snow and Landscape Research WSL, Zürcherstrasse 111, 8903 Birmensdorf, Switzerland
[4]Department of Life Sciences, Imperial College London, Silwood Park Campus, Buckhurst Road, Ascot SL5 7PY, UK
[5]Centre for Tropical Environmental Sustainability Studies, James Cook University, Cairns, QLD, 4878, Australia
[6]FFCLRP, Department of Biology, University of São Paulo, Ribeirão Preto, Brazil
[7]Department of Biological Sciences, Macquarie University, North Ryde, NSW 2109, Australia
[8]Department of Earth System Science, Tsinghua University, Beijing 100084, China

Corresponding Author: I. Colin Prentice (c.prentice@imperial.ac.uk)

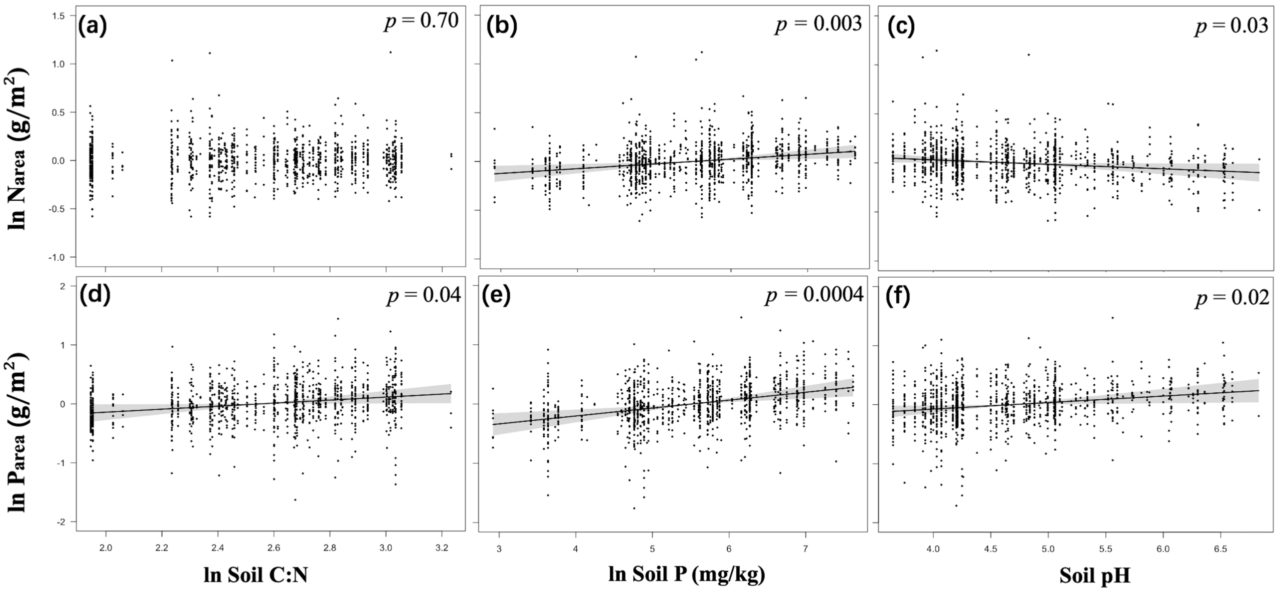

Fig. S1 Partial residual plots for leaf traits (all-species) in relation to *in situ* measured soil properties. Coefficients and standard errors for the fitted lines are given in Supporting Information Table S4.

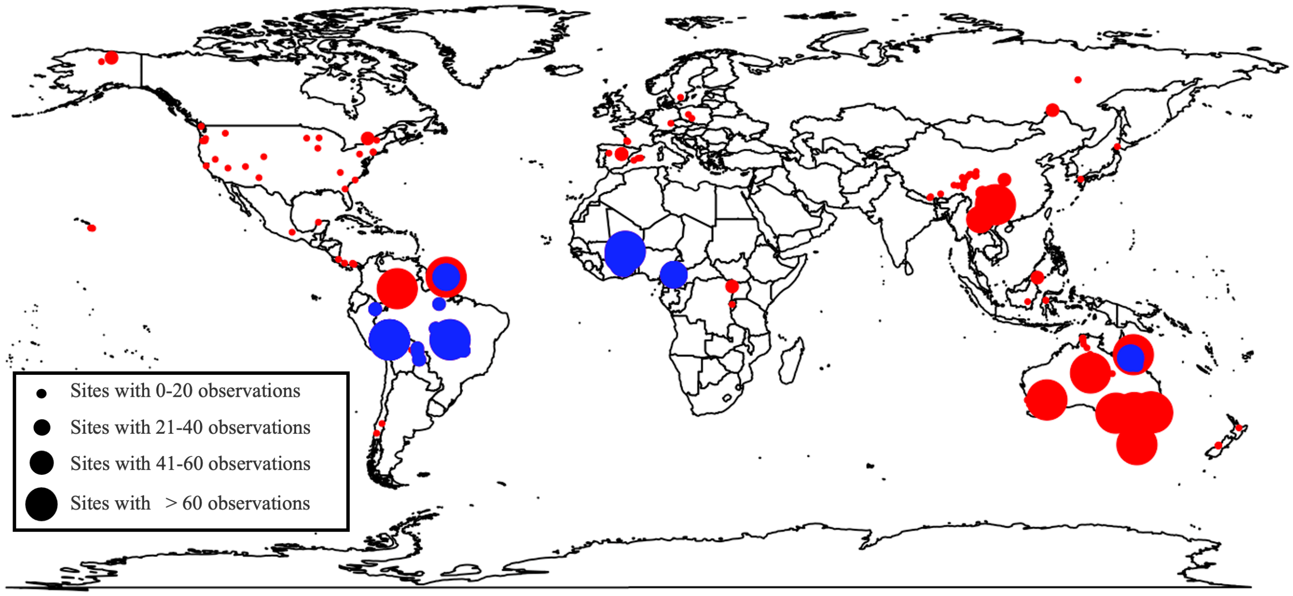

Fig. S2 Geographic distribution of sampling sites. The area coverage was scaled by number of observations at each site. Sites with *in situ* soil measurements are in blue; others in red.

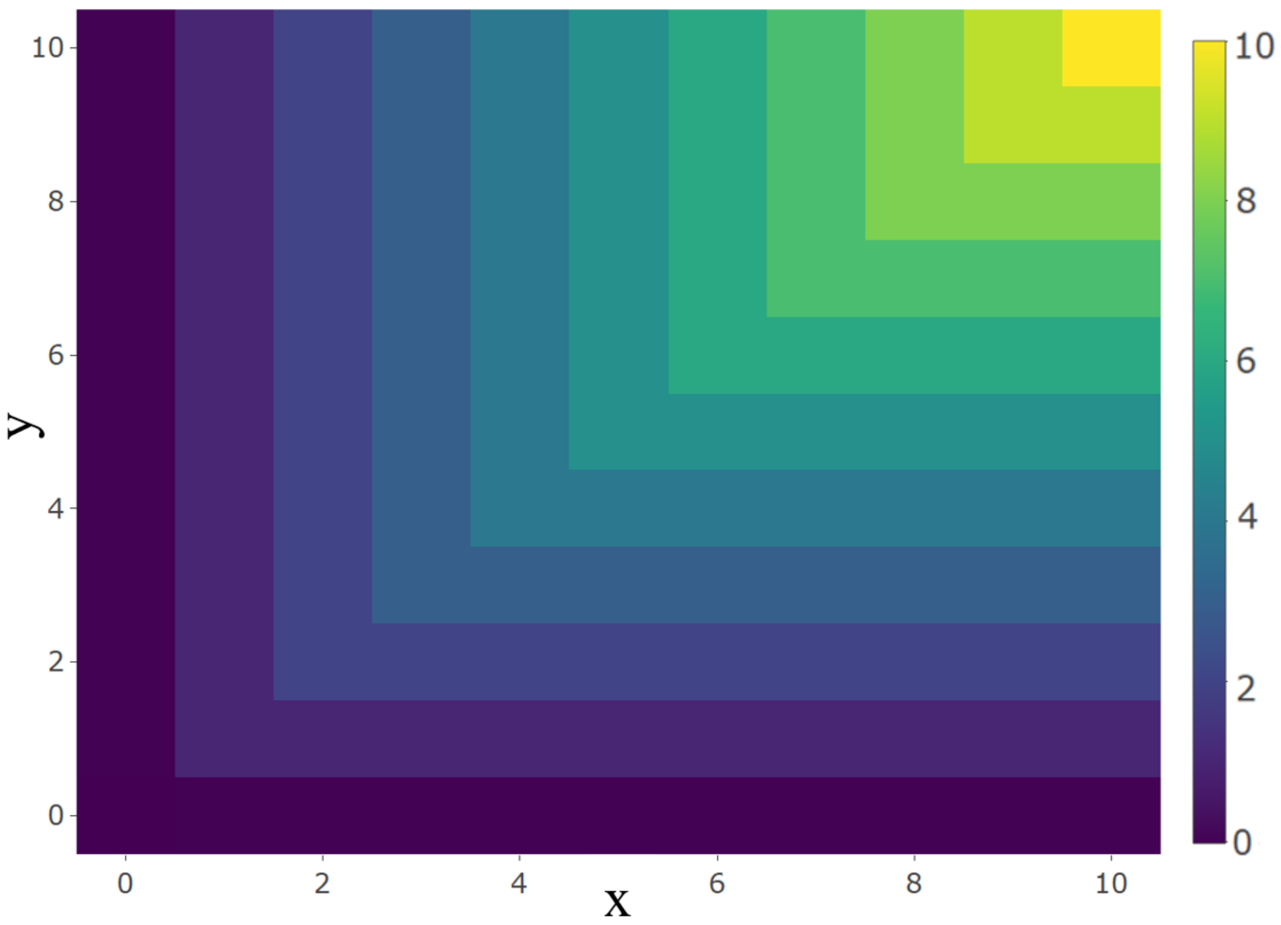

Fig. S3 The response of variable Z to the predictor variables x and y according to the minimum model represented by equation (12), with $k = 10$.

Table S1 Partitioning of trait variation unexplained by fixed effects. Iterative mixed-effects models used a common structure: with climate, leaf or soil trait components as the fixed terms (Figs 1-3 and S1) and a crossed-random design, which provides random intercepts for individual species and sites.

Response trait<br>Units		V_{cmax25}<br>μmol m^{-2} s^{-1}	Bias<br>%	Bias<br>%	N_{area}<br>g m^{-2}	P_{area}<br>g m^{-2}
Explanatory factor		Climates	Climates	Nutrients	Soil	Soil
Figure No.		1	2	3	S1	S1
Random component:		%	%	%	%	%
Species		22	20	20	37	12
Site		50	42	41	16	40
Residual		28	38	39	47	48

Table S2 Summary of linear regressions and minimum-function regressions between area-based photosynthetic capacity (V_{cmax25}; μmol m^{-2} s^{-1}) and leaf traits (N_{area} and P_{area}; g m^{-2}), for log-transformed site-mean and site-species data. Analysis of site-mean data was conducted by Ordinary least squares multiple linear regression (lm). Analysis of site-species data was carried out by a linear mixed-effects regression (lmer), with sites and species as random intercepts. An interactive model of V_{cmax25} versus N_{area} and P_{area} was provided in site-mean and all-species data separately. A non-linear minimum function model (nlm) of N_{area} and P_{area} for predicting V_{cmax25} was also conducted in both site-mean and all-species data (see 'log-sum-exp' formula in Equation 12).

**lm (site-mean)**	**a**	**b**	**c**	**d**	**r² (lm)**	**r² (nlm)**
$V_{cmax25} = a+b*N_{area}$	3.84^{***}	0.21^{*}			0.015	
$V_{cmax25} = a+b*P_{area}$	4.54^{***}	0.28^{***}			0.099	
$V_{cmax25} = a+b*N_{area}+ c*P_{area}$	4.43^{***}	0.12	0.27^{***}		0.104	**0.121**
$V_{cmax25} = a+b*N_{area}+c*P_{area}+d*N_{area}:P_{area}$	4.54^{***}	–0.04	0.32^{**}	–0.08	0.105	
**lmer (all-species)**	**a**	**b**	**c**	**d**	**r² (lmer)**	**r² (nlm)**
$V_{cmax25} = a+b*N_{area}$	3.73^{***}	0.25^{***}			0.035	/
$V_{cmax25} = a+b*P_{area}$	4.16^{***}	0.13^{***}			0.017	/
$V_{cmax25} = a+b*N_{area}+ c*P_{area}$	3.89^{***}	0.21^{***}	0.07^{***}		0.038	**0.050**
$V_{cmax25} = a+b*N_{area}+c*P_{area}+d*N_{area}:P_{area}$	3.88^{***}	0.23^{***}	0.06^{**}	0.01	0.038	

Table S3 Number of species recorded in each plant functional type within the leaf trait measurements dataset. The classification of species to plant functional types was performed using the TRY Categorical Traits Dataset (https://www.try-db.org/).

**Plant functional type**	**No. species**
Angiosperm evergreen trees	816
Angiosperm deciduous trees	191
Angiosperm evergreen shrubs	289
Angiosperm deciduous shrubs	127
Graminoids	27
Forbs	136
Pteridophytes	8
Gymnosperm evergreen trees	34
Gymnosperm deciduous trees	4
Gymnosperm evergreen shrubs	5

Table S4 Summary statistics for the relationships shown in Figs 1–4 and S1. All variables were log-transformed except T_g and soil pH. All-species analyses: degrees of freedom (Df) = 2509 (full data set) or 1188 (subset with *in situ* soils data). Site-mean analyses: Df = 262 (full data set) or 101 (subset with *in situ* soils data).

**Predictor of V_{cmax25}**	Coefficient	Std. Error	t value	*P*	Fig. No.
Intercept	-1.13	1.37	-0.82	0.41	
PPFD	0.99	0.22	4.49	<0.001	1a
T_g (K^{-1})	-0.04	0.01	-7.83	<0.001	1b
D	0.13	0.06	1.93	0.05	1c
R-Squared: 0.168	Df: 2509				
**Predictor of V_{cmax25}**					
Intercept	-1.20	1.31	-0.92	0.36	
PPFD	1.02	0.21	4.88	<0.001	1d
T_g (K^{-1})	-0.04	0.01	-8.48	<0.001	1e
D	0.13	0.06	2.06	0.04	1f
R-Squared: 0.314	Df: 262				
**Predictor of Bias**					
Intercept	14.56	50.21	0.29	0.77	
PPFD	0.32	8.08	0.04	0.97	2a
T_g (K^{-1})	-0.14	0.17	-0.78	0.44	2b
D	-3.65	2.35	-1.56	0.12	2c
R-Squared: 0.017	Df: 2509				
**Predictor of Bias**					
Intercept	7.66	39.23	0.20	0.85	
PPFD	0.62	6.30	0.10	0.92	2d
T_g (K^{-1})	-0.08	0.14	-0.56	0.57	2e
D	-3.31	1.92	-1.72	0.09	2f
R-Squared: 0.034	Df: 262				
**Predictor of Bias**					
Intercept	3.37	1.99	1.70	0.09	
N_{area}	-6.35	0.94	-6.77	<0.001	3a
P_{area}	-1.74	0.68	-2.58	0.01	3b
R-Squared: 0.035	Df: 2509				
**Predictor of Bias**					
Intercept	-2.85	3.19	-0.89	0.37	
N_{area}	-3.95	2.29	-1.73	0.09	3c
P_{area}	-3.50	1.23	-2.85	0.005	3d
R-Squared: 0.048	Df: 263				
**Predictor of N_{area}**	Coefficient	Std. Error	t value	*P*	Fig. No.
Intercept	0.84	0.25	3.37	0.001	
C:N	-0.02	0.06	-0.38	0.70	4a
Total P	0.05	0.02	2.47	0.02	4b

pH	-0.05	0.02	-2.23	0.03	4c
R-Squared: 0.108	Df: 101				
**Predictor of N_{area}**					
Intercept	0.64	0.22	2.95	0.004	
C:N	0.02	0.05	0.39	0.70	S1a
Total P	0.05	0.02	3.03	0.003	S1b
pH	-0.05	0.02	-2.14	0.03	S1c
R-Squared: 0.032	Df: 1188				
**Predictor of P_{area}**					
Intercept	-4.13	0.50	-8.24	<0.001	
C:N	0.25	0.12	2.00	0.05	4d
Total P	0.14	0.04	3.65	<0.001	4e
pH	0.11	0.05	2.44	0.02	4f
R-Squared: 0.163	Df: 101				
**Predictor of P_{area}**					
Intercept	-4.20	0.49	-8.53	<0.001	
C:N	0.26	0.12	2.08	0.04	S1d
Total P	0.13	0.04	3.66	<0.001	S1e
pH	0.11	0.05	2.36	0.02	S1f
R-Squared: 0.084	Df: 1188				

Table S5 Model performance: comparisons using different k values in Eq. 12.

**Site-mean**	**Intercept [N_{area}]**	**Slope [N_{area}]**	**Intercept [P_{area}]**	**Slope [P_{area}]**	**r^2**
$k = 5$	4.00	1.24	4.61	0.31	0.11
$k = 10$	3.91	1.09	4.62	0.31	0.12
$k = 20$	3.90	1.00	4.62	0.32	0.12
$k = 50$	3.92	0.91	4.62	0.32	0.12
$k = 100$	3.92	0.89	4.63	0.32	0.12
**All-species**	**Intercept [N_{area}]**	**Slope [N_{area}]**	**Intercept [P_{area}]**	**Slope [P_{area}]**	**r^2**
$k = 5$	3.72	0.28	4.68	0.32	0.04
$k = 10$	3.68	0.27	4.54	0.30	0.05
$k = 20$	3.67	0.26	4.50	0.30	0.05
$k = 50$	3.67	0.26	4.48	0.29	0.05
$k = 100$	3.66	0.26	4.47	0.29	0.05
