# Supplementary material for: Global climate and nutrient controls of photosynthetic capacity
Source: Commun Biol. 2021 Apr 12;4:462. doi: 10.1038/s42003-021-01985-7 (PMC8042000; doi:10.1038/s42003-021-01985-7)
Supplement: Supplementary file 3 — Reporting Summary [file 42003_2021_1985_MOESM3_ESM.pdf]

nature research

Corresponding author(s): Colin Prentice
Last updated by author(s): Feb 19, 2020

Reporting Summary

Nature Research wishes to improve the reproducibility of the work that we publish. This form provides structure for consistency and transparency in reporting. For further information on Nature Research policies, see our Editorial Policies and the Editorial Policy Checklist.

Statistics

For all statistical analyses, confirm that the following items are present in the figure legend, table legend, main text, or Methods section.

n/a	Confirmed	
☐	☒	The exact sample size (n) for each experimental group/condition, given as a discrete number and unit of measurement
☐	☒	A statement on whether measurements were taken from distinct samples or whether the same sample was measured repeatedly
☐	☒	The statistical test(s) used AND whether they are one- or two-sided<br>*Only common tests should be described solely by name; describe more complex techniques in the Methods section.*
☐	☒	A description of all covariates tested
☐	☒	A description of any assumptions or corrections, such as tests of normality and adjustment for multiple comparisons
☐	☒	A full description of the statistical parameters including central tendency (e.g. means) or other basic estimates (e.g. regression coefficient) AND variation (e.g. standard deviation) or associated estimates of uncertainty (e.g. confidence intervals)
☐	☒	For null hypothesis testing, the test statistic (e.g. F, t, r) with confidence intervals, effect sizes, degrees of freedom and P value noted<br>*Give P values as exact values whenever suitable.*
☒	☐	For Bayesian analysis, information on the choice of priors and Markov chain Monte Carlo settings
☒	☐	For hierarchical and complex designs, identification of the appropriate level for tests and full reporting of outcomes
☐	☒	Estimates of effect sizes (e.g. Cohen's d, Pearson's r), indicating how they were calculated

Our web collection on statistics for biologists contains articles on many of the points above.

Software and code

Policy information about availability of computer code

Data collection	The leaf traits measurement dataset was collected from several literatures shown in Method section. No new samples were collected in this study.
Data analysis	The statistical analysis has applied linear regression for site-mean analysis (n = 266) and mixed-effects model for all-species analysis (n = 2513), where sites and species were random intercept. Details of data collection, calculations, analyses were available in Methods. The additional statistics of main analyses were presented in Supporting Information.

For manuscripts utilizing custom algorithms or software that are central to the research but not yet described in published literature, software must be made available to editors and reviewers. We strongly encourage code deposition in a community repository (e.g. GitHub). See the Nature Research guidelines for submitting code & software for further information.

Data

Policy information about availability of data

All manuscripts must include a data availability statement. This statement should provide the following information, where applicable:
- Accession codes, unique identifiers, or web links for publicly available datasets
- A list of figures that have associated raw data
- A description of any restrictions on data availability

No new data were collected for this analysis. The photosynthesis, leaf-trait and soils data are available from the authors of papers cited in the Methods section7,8,14,20,28,73,89-94. The complete photosynthesis, climate, leaf-trait and soils datasets underlying all analyses are also publicly available at https://github.com/yunkepeng/VcmaxMS. In case of any issues concerning the observed and predicted data, and for all queries on ancillary information including the climate data, please contact Yunke Peng (yunke.peng@usys.ethz.ch) or Colin Prentice (c.prentice@imperial.ac.uk).

Field-specific reporting

Please select the one below that is the best fit for your research. If you are not sure, read the appropriate sections before making your selection.

☐ Life sciences ☐ Behavioural & social sciences ☒ Ecological, evolutionary & environmental sciences

For a reference copy of the document with all sections, see nature.com/documents/nr-reporting-summary-flat.pdf

Ecological, evolutionary & environmental sciences study design

All studies must disclose on these points even when the disclosure is negative.

Study description	We have collected a large leaf traits dataset including maximum rate of carboxylation capacity at 25 degree celcius (Vcmax25), leaf nitrogen per area (Narea) and leaf phosphorus per area (Parea), as well as a subset of dataset including measured soil traits: soil pH, soil C/N, and total soil Phosphorus. We also extracted climate and plant functional type data paralleled to all measured sites.
Research sample	The global leaf traits dataset has included 266 sites, 1637 species and 5000 individuals, and paralleled soil measurements for 105 sites. No new field measurements data were used in this database, and original source of this dataset was all available in Method Section.
Sampling strategy	We only selected samples that at least including measured Vcmax25, Narea and Parea. Where necessary, we have recorded its coordinates information, species name and measured soil data that have been furtherly used in our statistical analysis.
Data collection	Yunke Peng has collected all dataset, and this dataset has consisted of all literature sources as mentioned in Method Section.
Timing and spatial scale	Literatures data were collected between 1980 to 2017, across global scale.
Data exclusions	The samples were only excluded if the any key leaf traits (i.e. Vcmax25, Narea, Parea) were missing.
Reproducibility	Data collections, calculations and statistical analyses were described in Method Section. The additional statistics summary were presented in Supporting Information. The complete photosynthesis, climate, leaf-trait and soils datasets underlying all analyses are also publicly available at https://github.com/yunkepeng/VcmaxMS.
Randomization	The species and sites were distributed in global scale.
Blinding	The dataset has binded many sub-datasets from different sources, as given in Method Section.

Did the study involve field work? ☐ Yes ☒ No

Reporting for specific materials, systems and methods

We require information from authors about some types of materials, experimental systems and methods used in many studies. Here, indicate whether each material, system or method listed is relevant to your study. If you are not sure if a list item applies to your research, read the appropriate section before selecting a response.

Materials & experimental systems

n/a	Involved in the study	
☒	☐	Antibodies
☒	☐	Eukaryotic cell lines
☒	☐	Palaeontology and archaeology
☒	☐	Animals and other organisms
☒	☐	Human research participants
☒	☐	Clinical data
☒	☐	Dual use research of concern

Methods

n/a	Involved in the study	
☒	☐	ChIP-seq
☒	☐	Flow cytometry
☒	☐	MRI-based neuroimaging
